# LsrB-based and temperature-dependent identification of bacterial AI-2 receptor

**DOI:** 10.1186/s13568-017-0486-y

**Published:** 2017-10-10

**Authors:** Yuxi Zhang, Kezong Qi, Yawei Jing, Jiakun Zuo, Jiangang Hu, Xiaolong Lv, Zhaoguo Chen, Rongsheng Mi, Yan Huang, Shengqing Yu, Xiangan Han

**Affiliations:** 1 Shanghai Veterinary Research Institute, The Chinese Academy of Agricultural Sciences (CAAS), 518 Ziyue Road, Shanghai, 200241 People’s Republic of China; 2 College of Animal Science and Technology, Anhui Agricultural University, Hefei, 230036 People’s Republic of China

**Keywords:** *Escherichia coli* BL21, *LuxS* mutant, LsrB, AI-2 receptor

## Abstract

The *luxS* gene is required for autoinducer-2 (AI-2) synthesis in many bacterial species. AI-2 is taken up by a specific receptor to regulate multiple bacterial activities. However, the lack of methods to identify AI-2 receptors has impeded investigations into the roles of AI-2. Here, a *luxS* mutant of *Escherichia coli* strain BL21 (DE3) was constructed (named BL21∆luxS), and the recombinant LsrB protein of *Salmonella enterica* was expressed in BL21∆luxS and BL21 cells, which were named LsrB (BL21∆luxS) and LsrB (BL21), respectively. The results of the activity of recombinant LsrB binding showed that LsrB (BL21) bound to endogenous AI-2 (produced from BL21 strain), while LsrB (BL21∆luxS) did not (as BL21∆luxS cannot produce AI-2). However, the results of recombinant LsrB binding showed that LsrB (BL21∆luxS) can bind exogenous AI-2, which was released from LsrB (BL21∆luxS) at 55 °C for 10 min, while LsrB (BL21) could not bind exogenous AI-2 (due to binding of endogenous AI-2 before). Furthermore, analysis of the thermal stability of AI-2 showed that that AI-2 activity was relatively high at incubation temperatures below 65 °C. These findings will be beneficial for screening of new AI-2 receptors in different bacterial species.

## Introduction

Cell–cell communication in bacteria is accomplished through the exchange of extracellular signalling molecules, called autoinducers, in a process termed quorum sensing (QS) (Pereira et al. 2013). Although most autoinducers are species specific, autoinducer-2 (AI-2) is considered to be a universal signalling molecule for interspecies communication that is synthesised by LuxS, an enzyme that is highly conserved and widespread in diverse bacteria (Han and Lu 2009; Pereira et al. 2013; Even-Tov et al. 2016).

The LuxS/AI-2 type QS system plays important roles in the regulation of bacterial bioluminescence, sporulation, competence, antibiotic resistance, biofilm formation, and virulence factor secretion (Xue et al. 2013; Han et al. 2013, 2015a, b). AI-2 is taken up by a specific membrane receptor transport system. Previous studies have identified several AI-2 receptors in various bacterial species, including LuxP in *Vibrio harveyi* (Chen et al. 2002), LsrB in *Salmonella typhimurium* (Miller et al. 2004) and RbsB in *Haemophilus influenza* (Armbruster et al. 2011).

Some bacterial species lack the AI-2 synthase gene *luxS* and are, therefore, not capable of producing AI-2 (De Keersmaecker et al. 2006; Rezzonico and Duffy 2008). However, such bacteria, including *Sinorhizobium meliloti* (Pereira et al. 2013) and *Riemerella anatipestifer* (Han et al. 2015a, b), might use endogenous AI-2 to regulate physiological functions. Furthermore, the lack of knowledge of the underlying molecular mechanisms of AI-2 recognition, signal transduction and/or processing continues to impede understanding of the function of AI-2 in any given species. Undoubtedly, the lack of appropriate screening procedures is a major drawback in the identification of AI-2 receptors in other bacterial species and impedes further investigations into the roles of AI-2. In this study, a set of criteria was established to identify functional AI-2 receptors using LsrB in *S. typhimurium*. These findings will be beneficial for future screening of AI-2 receptors in different bacterial species.

## Materials and methods

### Bacterial strains, plasmids, and culture conditions

*Escherichia coli* strains DH5α and BL21 (DE3) (Invitrogen Corporation, Carlsbad, CA, USA) were grown at 37 °C in Lennox broth (LB) or on solid medium containing 1.5% agar at 37 °C and used for the cloning and expression of recombinant genes. When necessary, LB medium was supplemented with an appropriate dosage of ampicillin (100 μg/ml) or kanamycin (100 μg/ml).

The expression vector pCold-TF was purchased from Takara Bio, Inc. (Shiga, Japan). Restriction enzymes were purchased from MBI Fermentas, Inc. (Waltham, MA, USA). *V. harveyi* strains BB170 (sensor^1−^ sensor^2+^) (ATCC BAA-1117)and BB152 (ATCC BAA-1119)were purchased from the American type culture collection (Manassas, VA, USA) and cultivated in modified autoinducer bioassay (AB) medium (Bassler et al. 1993). BB170 was used as the AI-2 biosensor strain and BB152 as a positive control for AI-2 production. All chemicals used were of analytical grade and purchased from Sigma-Aldrich Corporation (St. Louis, MO, USA).

### Construction and identification of luxS mutant strain BL21∆luxS

**Fig. 1:**
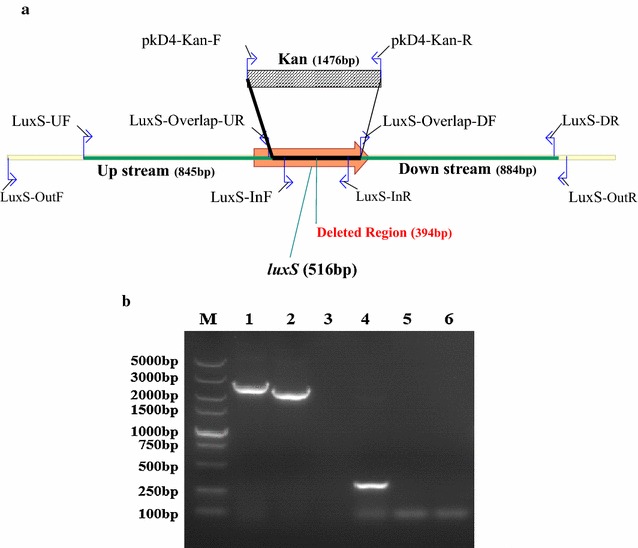
Identification of the *luxS* mutant BL21∆*luxS.* **a** Schematic chart of strategy for producing the BL21 *luxS* deletion mutant. The *luxS* was deleted by replacing the partial gene sequence of *luxS* with kanamycin resistance cassette at *Sal*I cleavage sites. The primers used for the confirmation of the *luxS* deletion are also indicated. **b** Identification of the *luxS* mutant BL21∆*luxS.* Lane M: DL 2000 DNA marker (D501A; Takara); lane 1: the wild-type strain BL21 showed a 2519-bp PCR product using primers LuxS-OutF/LuxS-OutR; lane 2: the mutant BL21∆*luxS* with cure of the kanamycin resistance cassette showed a 2209-bp PCR product using primers LuxS-OutF/LuxS-OutR; lane 3: negative control; lane 4: the wild-type strain DE17 showed a 307-bp PCR product using primers LuxS-inF/LuxS-inR; lane 5: the mutant DE17∆*pfs* showed no PCR products using primers LuxS-inF/LuxS-inR; lane 6: negative control

The upstream and downstream fragments (845 and 884 bp, respectively) of the BL21 (DE3) *luxS* gene were amplified by PCR using the primer pairs LuxS-UF/LuxS-Overlap-UR and LuxS-Overlap-DF/LuxS-DR (Table 1). The mutant strain BL21∆luxS was generated by creating a 394-bp in-frame deletion in the 516-bp *luxS* open reading frame using the lambda red recombination system (Fig. 1a), as described previously with slight modifications (Datsenko and Wanner 2000). Briefly, the upstream and downstream fragments of the *luxS* gene were ligated by overlap PCR using the primer pair LuxS-UF/LuxS-DR (Table 1) to produce a 1729-bp PCR product, and then the PCR product was sub-cloned into the pMD19-T vector to construct the recombinant plasmid pMD19-Up-Down. A kanamycin resistance cassette (Kan) was amplified from plasmid pKD4 by PCR using the primer pair pkD4-Kan-F/pkD4-Kan-R (Table 1). Then, the Kan was digested with the nuclease *Sal*I and subsequently inserted into the recombinant plasmid pMD19-Up-Down to form the plasmid pMD19-Up-Kan-Down. The mutagenic construct (containing the insertion sequence of Kan within the *luxS* gene coding region) was amplified by PCR using the primer pair LuxS-UF/LuxS-DR. Subsequently, the PCR product (3155 bp) was purified and used for electroporation. One microgram of PCR product was added to 100 μl of BL21 (DE3) competent cells containing the lambda red recombinase expression plasmid pKD46, and electroporation was performed using a Gene Pulser II transfection apparatus (Bio-Rad Laboratories, Hercules, CA, USA) at 25 μF, 2.4 kV and 250 Ω. After the electric pulse, the cells were diluted immediately in 1 ml of SOC (super optimal broth with catabolite repression) medium, incubated at 37 °C for 2 h and then plated on LB plates containing 100 μg/ml of Kan. After a 24-h incubation, the resulting Kan-resistant colonies were selected for PCR amplification with the primer pairs LuxS-inF/LuxS-inR and LuxS-OutF/LuxS-outR (Table 1) to identify the deletion of the *luxS* gene from BL21 cells. The mutant strain was transformed further with the pCP20 plasmid to cure the Kan and produce a Kan-sensitive mutant strain that was named BL21∆luxS.

**Table 1:** Primers used in this study

Primers	Oligonucleotide sequence (5′–3′)	Description	Product size (bp)
LuxS-UF	TTCTGATGCGCTGTTACGT	The upstream sequence of *luxS*	845
LuxS-Overlap-UR	TCGGCAGTGCCGC**GTCGAC**GGGGTGTTCATTGTTTTCG^a^		
LuxS-Overlap-DF	TGAACACCCC**GTCGAC**GCGGCACTGCCGAAAGAGAAG	The downstream sequence of *luxS*	884
LuxS-DR	TGACCGACGATAACCCGA		
pkD4-Kan-F	CGC**GTCGAC**TGTAGGCTGGAGCTGCTT	Kanamycin resistance cassette	1494
pkD4-Kan-R	CGC**GTCGAC**CATATGAATATCCTCCTTAGTTC		
LsrB-F	CGC**GGATCC**ATGGCAAGACACAGCATTAAAAT^b^	*lsrB*	1023
LsrB-R	CCC**AAGCTT**TCAGAAATCATATTTGTCGATATTG^c^		
LuxS-F	CGC**GGATCC**ATGCCGTTGTTAGATAGCTT^b^	*luxS*	516
LuxS-R	CCC**AAGCTT**CTAGATGTGCAGTTCCTGCA^c^		
LuxS-OutF	GCGATTTGTTCTTCTTTCCTG^d^	Primers for identification of *luxS* deletion	2519
LuxS-OutR	GATCAAGAATCGTCACAGG		
LuxS-InF	GAAGTGATGCCAGAAAGAGGG^e^	Primers for identification of *luxS* deletion	307
LuxS-InR	CCAGAATGCTACGCGCAATAT		

^a, b, c^ *Sal*I, *Bam*HI and *Hin*dIII restriction sites are underlined, respectively

^d^A 307 bp PCR product was amplified from wild-type strain BL21(DE3), and no product was amplified from mutant strain BL21Δ*luxS*, using primers LuxS-inF/LuxS-inR

^e^A 2519 or 2209 bp PCR product was amplified from wild-type strain BL21(DE3), or mutant strain BL21Δ*luxS* without kan, respectively, using primers LuxS-OutF/LuxS-OutR

### AI-2 assay

AI-2 activity is expressed in relative light units (RLU). The AI-2 bioassay was performed according to a previously described method with some modifications (Bassler et al. 1993; Han and Lu 2009; Han et al. 2013). Cell-free culture fluid (CF) was prepared as follows: *E. coli* strains BL21 (DE3), BL21∆luxS and DH5a were grown in LB at 37 °C, and then pelleted by centrifugation at 12,000*g* at 4 °C for 10 min. Then, the resulting supernatants were filtered through a 0.22-μm filter (EMD Millipore, Bedford, MA, USA) to obtain CF samples. The reporter strain *V. harveyi* BB170 was diluted to 1:5000 in fresh AB medium and then 180 μl of bacterial culture was mixed with 20 μl of the CF sample and incubated at 30 °C for 4 h. After incubation, 100-μl aliquots were added to white, flat-bottomed, 96-well plates (Thermo Labsystems, Franklin, MA, USA) for detection of AI-2 activity. A positive control was obtained from overnight cultures of BB152, while the CF from *E. coli* DH5a was used as a negative control. Luminescence was measured with a Tecan GENios Plus microplate reader in luminescence mode (TECAN Austria GmbH, Grödig, Austria). The AI-2 activity in CF of *V*. *harveyi* BB170 is reported as RLU. All samples were assayed in triplicate.

### Influence of temperature on AI-2 activity

To investigate the influence of temperature on AI-2 activity, AI-2 (Omm Scientific, Inc., Dallas, TX, USA) was dissolved in phosphate-buffered saline to form different concentrations of AI-2 solution (2.5, 10.0 and 40.0 μM). Then, the AI-2 solution was incubated for 10 min at 37, 45, 50, 55, 60, 65 or 70 °C, respectively. After a 10-min incubation, the AI-2 solution was filtered through a 0.22-μm filter (EMD Millipore) to obtain cell-free CF for detection of AI-2 activity. The AI-2 bioassay was performed as previously described (Han et al. 2013; Han et al. 2015a, b). All samples were assayed in triplicate.

### Expression and purification of recombinant LsrB and LuxS

The *lsrB* gene of *S. enterica* (NC_003198.1) and *luxS* gene of *E. coli* strain BL21 (DE3) (WP_001130211.1) were amplified using the oligonucleotides LsrB-F/LsrB-R and LuxS-F/LuxS-R, respectively (Table 1). The primers generated *Bam*HI and *Hin*dIII restriction site (underlined) that were used to clone the *Bam*HI/*Hin*dIII-digested PCR fragments into a *Bam*HI/*Hin*dIII-digested pCold TF vector to construct the two expression plasmids pColdTF-lsrB and pColdTF-luxS. *E. coli* strains BL21 and BL21∆luxS were transformed with the expression vectors pColdTF-lsrB and pColdTF-luxS, respectively, and grown on LB agar plates containing ampicillin (100 μg/ml). The *E. coli* strain BL21 containing the plasmid pColdTF-lsrB or pColdTF-luxS was named BL21 (pColdTF-lsrB) or BL21 (pColdTF-luxS), respectively. The *E. coli* strain BL21∆luxS containing the plasmid pColdTF-lsrB was named BL21∆luxS (pColdTF-lsrB). The addition of 1 mM isopropyl β-d-1-thiogalactopyranoside induced the expression of recombinant LsrB or recombinant LuxS. The recombinant LsrB of *S. enterica* was expressed in BL21∆luxS and BL21 *E*. *coli* cells, which were named LsrB (BL21∆luxS) and LsrB (BL21), respectively. Recombinant LsrB and LuxS proteins were isolated and purified using a HisTrap™ column according to the manufacturer’s protocol (Amersham Biosciences Corporation, Amersham, UK). The final protein concentration was determined by the Bradford method using the SmartSpec™ 3000 Spectrophotometer (Bio-Rad Laboratories). Bovine serum albumin was used as a standard.

### Binding of recombinant LsrB to AI-2

To determine whether recombinant LsrB can bind to endogenous AI-2, LsrB (BL21) and LsrB (BL21∆luxS) proteins were isolated from *E.coli* strain BL21 (pColdTF-lsrB) or BL21∆luxS (pColdTF-lsrB), respectively. Purified LsrB (BL21) and LsrB (BL21∆luxS) proteins (5 mg/ml) were incubated for 10 min at 37, 50 and 60 °C to release endogenous AI-2, respectively. After incubation, the LsrB proteins were removed by ultrafiltration (10,000-Da cut-off; EMD Millipore), and the filtered reaction products were tested for AI-2 activity using a *V. harveyi* BB170 bioassay, performed essentially as described above. The LuxS protein was isolated from BL21 (pColdTF-lsrB) cells as a negative control and AI-2 (10 μM) was used as a positive control. All samples were assayed in triplicate.

Different concentrations of exogenous AI-2 (2.5, 10.0 and 40.0 μM) were incubated with 5 mg/ml of LsrB (BL21∆luxS) protein for 10 min at 37 °C. After incubation, the LsrB (BL21∆luxS) protein was removed by ultrafiltration (10,000-Da cut-off, EMD Millipore) and the filtered reaction products were tested for AI-2 activity by investigating the binding activity of LsrB (BL21∆luxS) to exogenous AI-2. Further, the isolated LsrB (BL21∆luxS) proteins (5 mg/ml) were incubated for 10 min at 37, 50 and 60 °C to release exogenous AI-2, respectively. The LsrB (BL21∆luxS) protein was denatured for 10 min at 100 °C and then also incubated with exogenous AI-2 for 10 min at 37 °C. Afterward, the filtered reaction products were tested for AI-2 activity, as described above, with a negative control. Increasing concentrations of AI-2 (2.5, 10.0, and 40.0 μM) were used as positive controls, respectively. All samples were assayed in triplicate.

### Statistical analysis

All statistical analyses were conducted using SPSS ver. 19.0 software (SPSS Inc., Chicago, IL, USA). One-way analysis of variance was used to identify differences in biofilm formation and gene transcription levels. A probability (*p*) value of < 0.05 was considered statistically significant.

## Results

### Identification of the BL21ΔluxS mutant strain

Two primer pairs, LuxS-OutF/LuxS-OutR and LuxS-inF/LuxS-inR, were used for PCR analysis (Fig. 1a) to confirm the deletion of the *luxS* gene from BL21 (DE3) cells. A 2519-bp PCR product was amplified from wild-type strain BL21 (DE3) (Fig. 1b, lane 1), while the mutant BL21∆luxS with cure of the kanamycin resistance cassette showed a 2209-bp PCR product using the primer pair LuxS-OutF/LuxS-OutR (Fig. 1b, lane 2). No product was amplified from the mutant strain BL21Δ*luxS* (Fig. 1b, lane 5), while a 307-bp (Fig. 1b, lane 4) PCR product was amplified from wild-type strain BL21 (DE3) using the primer pair LuxS-InF/LuxS-InR.

### The BL21ΔluxS mutant strain lacked AI-2 activity

**Fig. 2:**
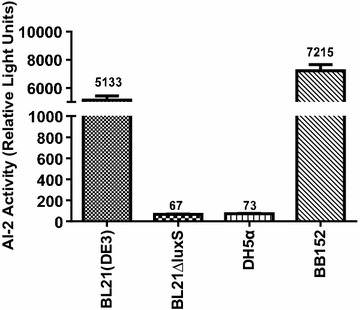
The AI-2 activity in BL21∆luxS. The wild strain BL21 secretes AI-2-like molecules, and can induce *V*. *harveyi* BB170 bioluminescence, whereas no bioluminescence induction was observed for the mutant BL21∆*luxS*. *V*. *harveyi* BB152 served as a positive control and *E. coli* DH5α as a negative control. The figure represents the means of the results from three independent experiments. The error bars indicate standard deviations

No AI-2 activity was detected from bacterial CF prepared from mutant strain BL21Δ*luxS,* in which the *luxS* gene was deleted (Fig. 2). However, *E*. *coli* wild-type strain BL21 and *V*. *harveyi* strain BB152 (positive control) both secrete AI-2-like molecules that can induce bioluminescence of *V*. *harveyi* strain BB170. The results showed that the *luxS* mutant BL21 (DE3) strain did not produce AI-2-like signalling molecules.

### Thermal stability of AI-2

**Fig. 3:**
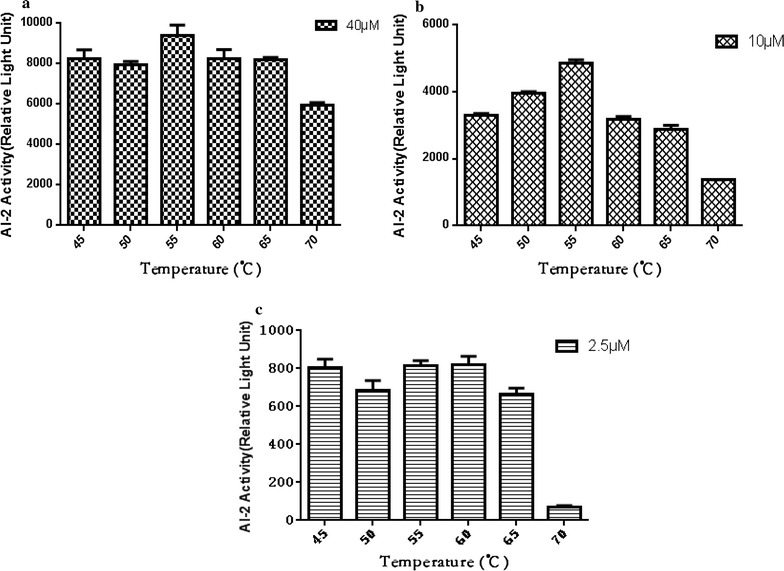
Thermal stability of AI-2. The different concentrations of AI-2 solution 40.0 μM (**a**), 10.0 μM (**b**) and 2.5 μM (**c**), respectively. Then, the AI-2 solution was incubated for 10 min at 37, 45, 50, 55, 60, 65 or 70 °C, respectively. After a 10-min incubation, the AI-2 solution was filtered through a 0.22-μm filter (EMD Millipore) to obtain cell-free CF for detection of AI-2 activity. AI-2 activity was expressed as relative light units (RLUs). The results of the thermal stability assay showed that AI-2 activity was relatively high at incubation temperatures of less than 65 °C. Furthermore, an incubation temperature of 45–65 °C had no significant effect on AI-2 activity. Experiments were repeated three times

The results of the thermal stability assay showed that AI-2 activity was relatively high at incubation temperatures of less than 65 °C. However, AI-2 activity was significantly reduced at 70 °C and decreased by 20% (Fig. 3a), 55% (Fig. 3b) and 90% (Fig. 3c), respectively, at concentrations of 2.5, 10.0 and 40.0 μM. Furthermore, an incubation temperature of 45–65 °C had no significant effect on AI-2 activity.

### Expression and purification of LsrB and LuxS

**Fig. 4:**
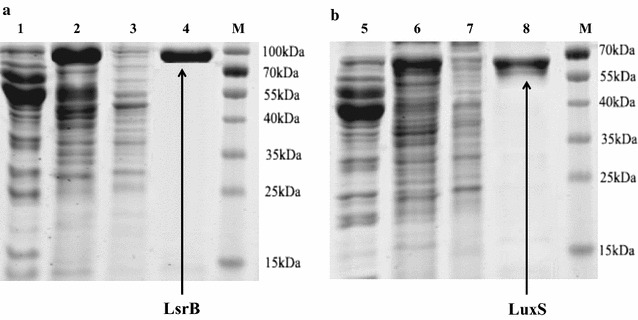
SDS-PAGE analysis of total cellular proteins and purified fusion proteins from BL21∆luxS cells. Lane 1 and lane 5: SDS-PAGE analysis of total cellular proteins from BL21∆luxS containing pCold TF (serve as negative control). Lane 2 and lane 6: SDS-PAGE analysis of total cellular proteins containing expression plasmids pCold-TF-lsrB (**a**) and pCold-TF-luxS (**b**), respectively. Lane 3 and lane 7: SDS-PAGE analysis of total precipitation proteins containing expression plasmids pCold-TF-lsrB and pCold-TF-luxS, respectively. Lane 4 and lane 8: elution of the LsrB and LuxS purified fusion protein from the affinity column, respectively

The recombinant vectors pColdTF-lsrB and pColdTF-luxS were expressed successfully in *E. coli* BL21 and BL21∆luxS cells. The transformant supernatants were analysed by sodium dodecyl sulphate polyacrylamide gel electrophoresis (SDS-PAGE) and the fusion proteins were clearly observed as bands with the expected molecular weights of 84.8 kDa (including a 48.0-kDa fusion protein tag) for LsrB and 57.4 kDa (including a 48.0-kDa fusion protein tag) for LuxS (Fig. 4).

The purity of the proteins was checked by 12% SDS-PAGE, which demonstrated single bands with the expected molecular weights (Fig. 4). The final concentrations of the LsrB and LuxS proteins were both 5 mg/ml.

### Binding of LsrB to endogenous AI-2

**Fig. 5:**
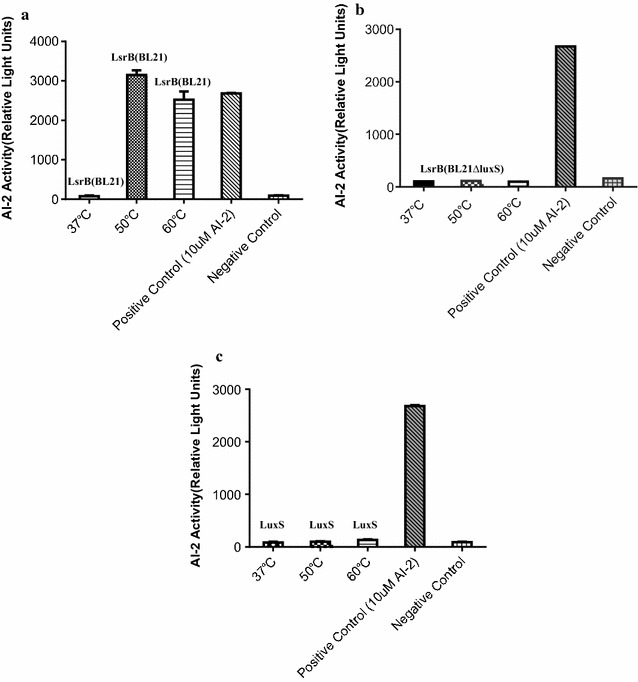
Binding of LsrB to endogenous AI-2. Purified LsrB (BL21) and LsrB (BL21∆luxS) proteins (5 mg/ml) were incubated for 10 min at 37, 50 and 60 °C to release endogenous AI-2, respectively. After incubation, the LsrB proteins were removed by ultrafiltration (10,000-Da cut-off; EMD Millipore), and the filtered reaction products were tested for AI-2 activity using a *V. harveyi* BB170 bioassay. The extent of LsrB binding to endogenous AI-2 was evaluated using an AI-2 assay, which showed that recombinant LsrB (BL21) bound to endogenous AI-2 (produced by wild-type strain BL21) and was released from LsrB (BL21) at 50 or 60 °C, respectively (**a**). However, since the *luxS* mutant BL21∆luxS did not produce endogenous AI-2, no AI-2 could be released from the recombinant LsrB (BL21∆luxS) (**b**). Moreover, the recombinant LuxS protein, which was expressed in strain BL21 (pColdTF-lsrB) as a negative control, also showed no AI-2 binding activity (**c**). AI-2 (10 μM) was used as a positive control

The extent of LsrB binding to endogenous AI-2 was evaluated using an AI-2 assay, which showed that recombinant LsrB (BL21) bound to endogenous AI-2 (produced by wild-type strain BL21) and was released from LsrB (BL21) at 50 or 60 °C, respectively (Fig. 5a). However, since the *luxS* mutant BL21∆luxS did not produce endogenous AI-2, no AI-2 could be released from the recombinant LsrB (BL21∆luxS) (Fig. 5b). Moreover, the recombinant LuxS protein, which was expressed in strain BL21 (pColdTF-lsrB) as a negative control, also showed no AI-2 binding activity (Fig. 5c). AI-2 (10 μM) was used as a positive control.

### Binding of LsrB to exogenous AI-2

**Fig. 6:**
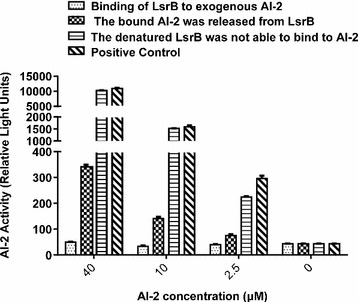
Binding of LsrB to exogenous AI-2. Different concentrations of AI-2 (2.5, 10.0 and 40.0 μM) were incubated with 5 mg/ml of LsrB (BL21∆luxS). The results of AI-2 binding activity indicated that 5 mg/ml of LsrB (BL21∆luxS) could bind exogenous AI-2 from 2.5 to 40 μM. Furthermore, the assay of AI-2 releasing activity showed that bound AI-2 was released from LsrB (BL21∆luxS) at 55 °C for 10 min. However, denatured LsrB (BL21∆luxS) was not able to bind to AI-2

Different concentrations of AI-2 (2.5, 10.0 and 40.0 μM) were incubated with 5 mg/ml of LsrB (BL21∆luxS). The results of AI-2 binding activity indicated that 5 mg/ml of LsrB (BL21∆luxS) could bind exogenous AI-2 from 2.5 to 40 μM (Fig. 6). Furthermore, the assay of AI-2 releasing activity showed that bound AI-2 was released from LsrB (BL21∆luxS) at 55 °C for 10 min (Fig. 6). However, denatured LsrB (BL21∆luxS) was not able to bind to AI-2.

## Discussion

AI-2, which was first identified in the marine bacterium *V. harveyi*, is produced by many Gram-negative and -positive bacteria (Surette et al. 1999). AI-2 is produced by the activated methyl cycle by the AI-2 synthase Pfs and LuxS. The LuxS-mediated QS system play roles in biofilm formation, virulence gene expression, the type III secretion system, and drug sensitivity changes in different bacterial species by AI-2 (Xue et al. 2013; Novotny et al. 2015; Mou and Plummer 2016). AI-2 is taken up by specific membrane receptors in bacterial transport systems to regulate physiological functions, such as LuxP in *V. harveyi* (Chen et al. 2002), LsrB in *S. typhimurium* (Miller et al. 2004) and RbsB in *H. influenza* (Armbruster et al. 2011). Furthermore, the AI-2 receptors also play roles in some host-microbial relationships. For example, an AI-2 mimic detected by the bacterial AI-2 receptor, LuxP/LsrB, can activate QS-controlled gene expression in the enteric pathogen *S. typhimurium* (Ismail et al. 2016). Therefore, further research of the AI-2 receptors will help to elucidate the mechanisms underlying the LuxS/AI-2 type QS system.

**Fig. 7:**
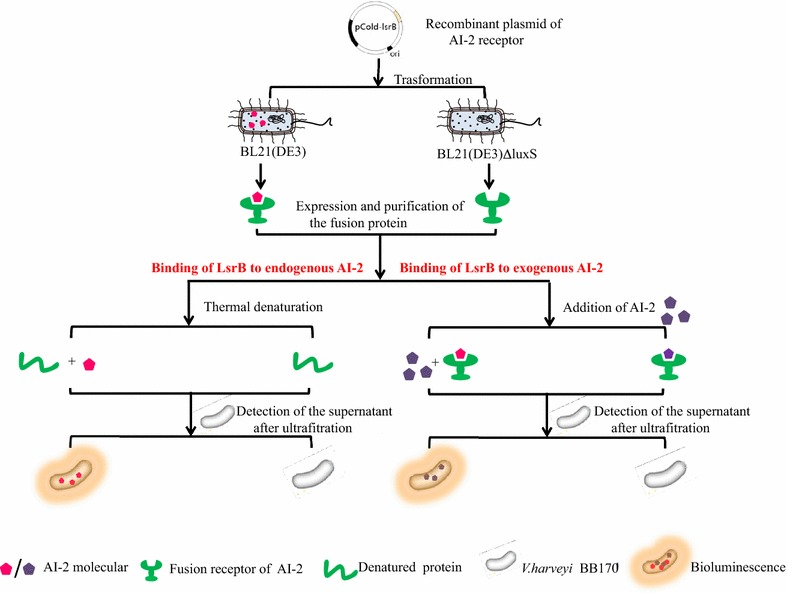
Schematic chart of strategy for binding of LsrB to endogenous AI-2 or exogenous AI-2. The extent of LsrB binding to endogenous AI-2 was evaluated using an AI-2 assay, which showed that recombinant LsrB (BL21) bound to endogenous AI-2 (produced by wild-type strain BL21) and was released from LsrB (BL21) at 50 or 60 °C. However, since the *luxS* mutant BL21∆luxS did not produce endogenous AI-2, no AI-2 could be released from the recombinant LsrB (BL21∆luxS). The combination of endogenous AI-2 produced by BL21 (DE3), which is known to interfere with the binding of AI-2 to LsrB (BL21), resulted in the loss in the ability of LsrB (BL21) to bind to exogenous AI-2. Hence, the BL21 (DE3) luxS mutant, which was incapable of producing endogenous AI-2, but could bind to exogenous AI-2

In this study, the combination of endogenous AI-2 produced by BL21 (DE3), which is known to interfere with the binding of AI-2 to LsrB (BL21), resulted in the loss in the ability of LsrB (BL21) to bind to exogenous AI-2. Hence, the BL21 (DE3) *luxS* mutant, which was incapable of producing endogenous AI-2, was constructed to express the recombinant protein LsrB and eliminate the interference from endogenous AI-2. Since the BL21∆luxS mutant eliminated endogenous AI-2, the LsrB (BL21∆luxS) mutant, which was overproduced in strain BL21∆luxS, had no AI-2 activity upon denaturation, but could bind to exogenous AI-2. However, the LsrB (BL21) overproduced in strain BL21 had AI-2 activity upon denaturation, but could not bind exogenous AI-2 (Fig. 7).

Research is needed on the discovery of molecules, like LsrB, that are AI-2 receptors and can release endogenous AI-2 upon denaturation or bind exogenous AI-2 when expressed in BL21 or BL21∆luxS, respectively. In the present study, the *luxS* gene of *E. coli* strain BL21 was expressed in BL21. However, the LuxS could not bind to endogenous or exogenous AI-2 (Fig. 5). The results confirmed that the proposed assay based on LsrB could be used for identification of new AI-2 receptors in future studies.

Furthermore, the present study also optimized the conditions of LsrB binding or releasing AI-2. Although AI-2 activity was high, an incubation temperature of 45–65 °C had no significant effect on AI-2 activity. Interesting, the results of AI-2 releasing activity showed that the optimal temperature for release of AI-2 bound to LsrB was 55 °C, within the range of 45–65 °C, and not affect the detection of AI-2 activity in *V*. *harveyi* BB170 bioluminescence. These findings will beneficial for future screening of new AI-2 receptors and to further study the roles of AI-2 in different bacterial species.

## Abbreviations

AI-2: autoinducer-2
QS: quorum sensing
LB: lennox broth
AB: autoinducer bioassay
Kan: kanamycin resistance cassette
CF: cell-free culture fluid
